# Electronic Spectroscopy of Monocyclic Carbon Ring Cations for Astrochemical Consideration

**DOI:** 10.1021/acs.jpca.2c00650

**Published:** 2022-03-28

**Authors:** Johanna Rademacher, Elliott S. Reedy, Ewen K. Campbell

**Affiliations:** School of Chemistry, University of Edinburgh, Edinburgh EH8 9YL, United Kingdom

## Abstract

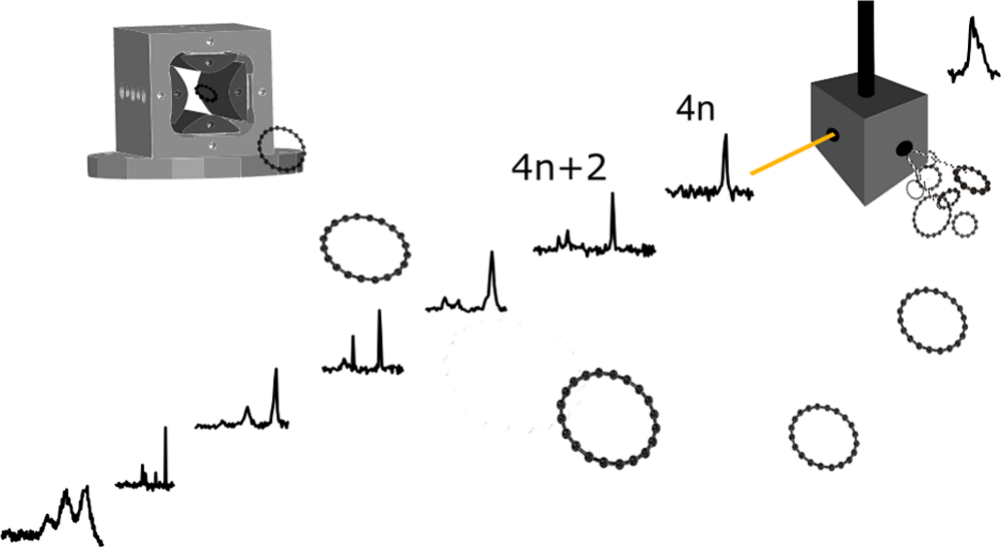

Gas phase electronic spectra of pure carbon cations generated by laser vaporization of graphite in a supersonic jet and cooled to below 10 K and tagged with helium atoms in a cryogenic trap are presented. The measured C_2*n*_^+^–He with *n* from 6 to 14, are believed to be monocyclic ring structures and possess an origin band wavelength that shifts linearly with the number of carbon atoms, as recently demonstrated through N_2_ tagging by Buntine et al. (J. Chem. Phys.2021, 155, 2143023487967910.1063/5.0070502). The set of data presented here further constrains the spectral characteristics inferred for the bare C_2*n*_^+^ ions to facilitate astronomical searches for them in diffuse clouds by absorption spectroscopy.

## Introduction

Since the detection of long polar chains in dense clouds in the 1970s,^1−4^ carbon containing molecules have played an important role in astrochemical studies. In contemporary work, the first carbon ring structures were identified by rotational spectroscopy; cyanobenzene, c-C_6_H_5_CN,^5^ and 1- and 2-cyanonaphthalene, C_10_H_7_CN.^6^ These possess large dipole moments so can be observed by radiotelescopes, and their detection is of relevance to the PAH hypothesis and thus the quest to reveal the nature of the species responsible from the so-called unidentified infrared bands (UIR) bands.^7^

Pure carbon structures are not amenable to detection by such methods, but they can be searched for based on their electronic transitions in absorption. This approach has been used to detect the spectroscopic fingerprints of the Buckminsterfullerene cation, C_60_^+^, in diffuse clouds.^8,9^ This soccer ball is the only identified species found to be responsible for a handful of diffuse interstellar bands (DIBs)^10−13^ and is remarkably abundant in these environments,^14^ presumably due to its resilience against destruction.

Other carbon isomers are also of interest in relation to the DIB enigma, but a lack of spectroscopic data has prevented evaluation of their astrochemical importance. This statement is particularly relevant for other open-shell C_*n*_^+^ cations^15^ which, due to their low ionization potentials,^16^ may be expected to be prevalent as for C_60_^+^ in environments bathed in photons with energies up to 13.6 eV. The versatile bonding of carbon leads to a variety of ionic structures ranging from linear chains to mono- and bicyclic rings and fullerenes, as revealed in ion mobility studies.^17−19^ The detection of their absorptions under appropriate conditions to enable comparison with astronomical data is an experimental challenge. As a result, little spectroscopic data has been added to that compiled in the reviews in the 1990s by van Orden and Saykally,^20^ and Bowers and colleagues,^18^ in which this knowledge gap was highlighted. To address this, approaches involving nonstandard methods for synthesis combined with cooling, and action spectroscopy in cryogenic traps have been recently applied.^21^

An instrument was constructed in our laboratory to combine laser vaporization synthesis with mass-selection and cryogenic ion trapping spectroscopy, and proof-of-principle results on helium tagged cyclic C_6_^+^ were presented.^22^ Various action spectroscopy methods were used to detect the ^2^Π_*g*_ ← *X*^2^Σ_*u*_^+^ electronic transition of linear C_5_^+^.^23^ In the latter study, spectra of C_5_^+^–He_*n*_ (*n* = 1–3) were used to predict the absorption wavelengths for the bare ion. This was proved by more direct 2-color experiments that monitored fragmentation of C_5_^+^ into C_3_^+^ + C_2_. Interestingly, a blue shift in the absorption wavelengths with *n* was observed, with a helium atom shifting the absorption bands of C_5_^+^ by around 10 cm^–1^. The same apparatus was used to spectroscopically show that the soccer ball isomer is the dominant structure with *m*/*z* = 720 produced by laser vaporization in the ion source.^24^

Buntine et al. very recently presented results using an instrument that combines ion mobility with spectroscopic characterization in a cylindrical ion trap at 25 K, reporting N_2_ tagged data of C_2*n*_^+^ monocyclic rings, with *n* = 6–14. These beautiful experiments are a breakthrough in attempts to attain the electronic spectra of carbon cations and reveal that the lowest energy doublet electronic transitions shift linearly to longer wavelengths with increasing *n*.^25^ The behavior thus resembles that observed for linear chains by Maier and colleagues, with examples being C_*n*_ and the neutral and positively charged polyacetylenes HC_*n*_H.^26^

Concerning the geometrical and electronic structure of cationic monocyclic rings, the only computational studies reported have used density functional theory (DFT) approaches.^27,28^ As noted elsewhere, such methods can be unreliable even for neutral carbon ring structures, leading to incorrect predictions of cumulenic/polyynic bonding.^29,30^ Recent studies suggest that a careful choice of functionals might be able to circumvent these problems and make DFT applicable to similar systems in the future.^31,32^ The open-shell nature of the C_2*n*_^+^ considered in the present study, however, require theoretical work using multireferential methods, but unfortunately no high level computations are currently available for these systems.

The C_2*n*_^+^ (*n* = 6–14) species reported in ref (25) are of interest in the context of the DIBs because their wavelength range (400–1400 nm) spans the region where the majority of them are found. The measured bandwidths show an alteration with 2*n*, and the narrowest absorptions have widths of 10–20 cm^–1^ in the visible, making them appealing targets for further laboratory and observational studies. In this contribution, the electronic spectra of helium tagged C_2*n*_^+^ (*n* = 6–14) are reported. These data are expected to be less perturbed by the tag and are reported to better constrain astronomical searches for these pure carbon structures.

## Experimental Section

Experiments were carried out using a recently constructed instrument described in detail in ref (22). Briefly, pulsed λ = 355 nm radiation (20 mJ/pulse) was focused onto a rotating and translating graphite rod producing C_2*n*_^+^ that were expanded in a supersonic jet of helium. After collimation with a skimmer, ions were transmitted through a quadrupole mass filter and turned through 90° before loading into the linear quadrupole ion trap, operating at a nominal (trap wall) temperature, *T*_nom_ = 4–5 K. Several pulses of ions, produced at 10 Hz, were accumulated in the trap and cooled through collisions with helium buffer gas, for several hundred milliseconds. Under these conditions, it was possible to convert a proportion of C_2*n*_^+^ into C_2*n*_^+^–He complexes. After pumping out the buffer gas, the trap contents were analyzed using a quadrupole mass-spectrometer coupled to a Daly detector.

Photofragmentation spectra were collected by exposing the stored ions to radiation from a pulsed, tunable OPO system supplying radiation over the 470–1340 nm range with a bandwidth of 5 cm^–1^. The OPO wavelength was calibrated using a wavemeter. Spectra were acquired with a step size of 0.1–0.4 nm at shorter wavelengths than 700 nm. At longer wavelengths, the minimum step size of 1 nm was used. On resonance with an electronic transition of C_2*n*_^+^–He, this led to dissociation of the complex by loss of the helium atom. To account for fluctuations in the number of stored ions, data were collected on alternate trapping cycles (repeated at 1 Hz) with (*N*_i_) and without (*N*_0_) exposure to the OPO, which was controlled by use of a mechanical shutter. By monitoring the attenuation (1 – *N*_i_/*N*_0_), C_2*n*_^+^–He photofragmentation spectra were obtained. The power of the OPO system was attenuated such that less than 30% of the C_2*n*_^+^–He were dissociated. The presented spectra are corrected for changes to the OPO power as a function of wavelength and are reported in air.

## Results and Discussion

### Electronic Spectra

**Figure 1:**
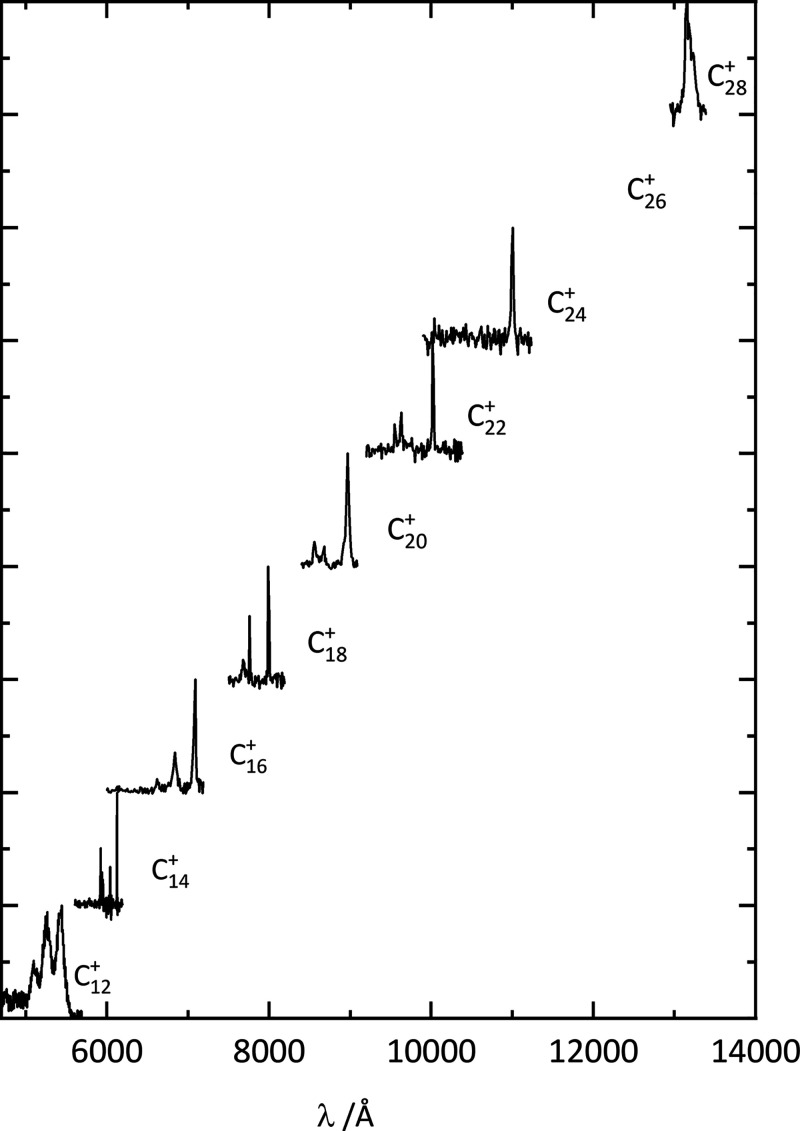
Electronic spectra of C_2*n*_^+^–He observed by monitoring the wavelength dependent loss of the helium atom. The data are believed to be the lowest energy doublet transitions of monocyclic cation ring structures.

The electronic spectra of C_2*n*_^+^–He (*n* = 6–14) complexes are presented in Figure 1, showing a linear increase in the wavelength of the origin bands with increasing ring size. A similar result for C_2*n*_^+^–N_2_ has been reported in ref (25) and provides confidence that the same molecular structures are probed in both experiments. The observed spectra are believed to be the lowest energy doublet electronic transitions of these open-shell carbon cations, and in most cases, the pattern is dominated by a strong origin band.

The assignment of these spectra to a series of monocyclic rings possessing similar structures suggests that information on the isomeric composition of C_2*n*_^+^ produced in the source can be obtained. To investigate this, the stored C_2*n*_^+^–He ions were irradiated with high fluence at the wavelength of their absorption bands shown in Figure 1. These experiments led to a maximum attenuation, and from this the number of C_2*n*_^+^–He remaining in the trap and having a different geometric structure can be estimated. The results of these attenuation experiments are listed in Table 1.

**Table 1:** Observed Maximum Attenuation  of C_*n*_^+^–He Complexes Following Irradiation at the Wavelength of Their Band Maxima Using High Fluence

C_*n*_^+^–He		
*n*	irradiation wavelength/Å	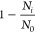
12	5439	0.97 ± 0.02
14	6123	0.96 ± 0.03
16	7088	0.97 ± 0.01
18	7988	0.93 ± 0.02
20	8968	0.96 ± 0.02
22	10017	0.67 ± 0.10
24	11007	0.60 ± 0.07
26		
28	13156	0.27 ± 0.14

Previous ion mobility experiments revealed that in the size range containing between 10 and 20 carbon atoms, cations with monocyclic ring structures are almost exclusively produced by laser vaporization of graphite.^18^ This is broadly consistent with the values presented in Table 1, based on spectroscopic measurement. For larger species, C_22_^+^–C_28_^+^, the number of complexes that do not interact with the radiation increases from about 30% for C_22_^+^ to ∼70% at C_28_^+^. Ion mobility studies indicate a significant reduction in % of monocycles should be expected in this region, where bicyclic structures become increasingly important. Note, however, that the number of C_2*n*_^+^ monocycles in the range *n* = 11–14 inferred in the present work diverges somewhat from the values presented in ref (18), where 50% of C_28_^+^ possess the same monocyclic ring structure. The differences between ion mobility and these spectroscopic results may be due to several reasons. For example, it is important to note that in the present work, the experiment is sensitive to the percentages of helium complexes depleted, which may not necessarily reflect the ratios of parent untagged C_2*n*_^+^ synthesized in the source. Different binding energies of the helium atoms for other structures, or relaxation processes that do not lead to the loss of the helium atom, could distort the isomeric picture inferred. Another possibility is that the conditions in the ions source (λ, fluence, pulse length/intensity) influence the distribution in this size range.

**Figure 2:**
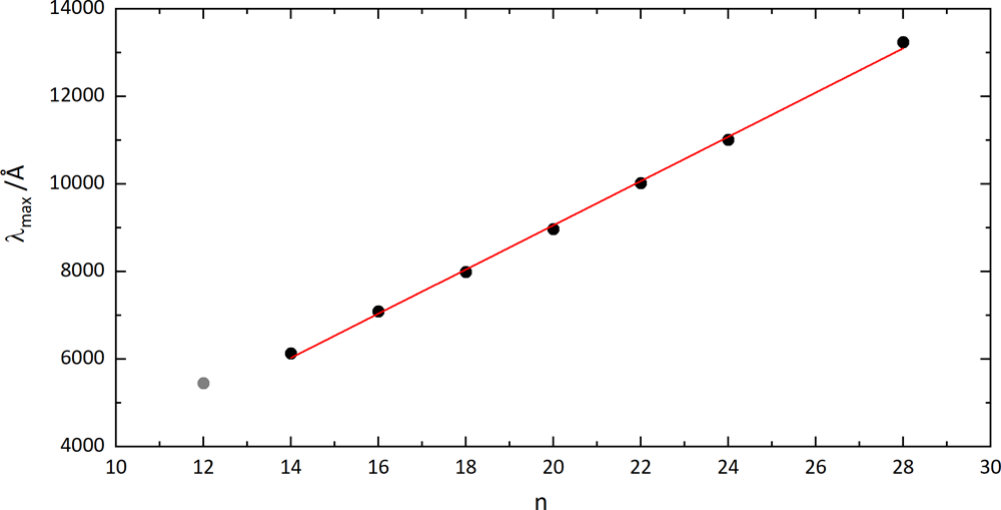
Wavelengths of origin band maxima as a function the number of carbon atoms (black). A linear fit to these wavelengths is the red line.

The wavelengths of the origin bands of the C_2*n*_^+^–He electronic transitions assigned to monocyclic rings are plotted in Figure 2, and they are also listed in Table 2. The profiles of the C_2*n*_^+^–He absorptions are asymmetric; therefore, band maxima are listed rather than the results of fits. The large step size in wavelength of the OPO coupled with the width/appearance of the narrowest features indicates that the origin band maxima are likely to fall between data points, particularly for the C_4*n*+2_^+^ spectra. The band maxima reported in Table 2 were estimated using the weighted average of the intensity of the data points closest to the one showing the strongest attenuation. For example, for C_18_^+^–He, the weighted average of three points gives 7991 Å compared with the data point at maximum intensity, 7988 Å. In comparison with the C_2*n*_^+^–N_2_ data reported in ref (25), the C_4*n*+2_^+^–He absorptions lie to the red and are shifted by between 3 and 26 cm^–1^. Both blue and red shifts are observed for the C_4*n*_^+^–He in comparison to the values listed in Table 1 of ref (25). Note also that the N_2_ tagged data were obtained at a higher trap temperature (25 K) than in the present work.

**Table 2:** Origin Band Maxima and fwhm’s for the Observed C_*n*_^+^–He Cations

C_*n*_^+^–He			C_*n*_^+^^c^
*n*	λ_max_^a^/Å	fwhm/Å	λ_max_/Å
12	5424 ± 10	76	5425 ± 10
14	6124 ± 1	2–3	6126 ± 2
16^b^	7086 ± 5	27	7088 ± 5
18	7991 ± 5	17	7995 ± 6
20	8968 ± 5	39	8972 ± 6
22	10020 ± 5	20	10025 ± 6
24	11005 ± 5	32	11012 ± 9
26			
28	13234 ± 7	60	13243 ± 10

a Reported uncertainties are  the OPO bandwidth for C_14_^+^, and  the step size for C_16_^+^ to C_24_^+^. The C_12_^+^ and C_28_^+^ characteristics are from Lorentzian fits to the observed data.

b Uncertain due to change from the signal to the idler at 710 nm.

c Predicted wavelengths for the untagged C_*n*_^+^ ions are based on extrapolation of C_*n*_^+^–He_1,2_ data shown in Figure 5 for C_14_^+^ and C_24_^+^. For the other C_*n*_^+^ ions, the average value of 5 cm^–1^ was used (see text for details).

**Figure 3:**
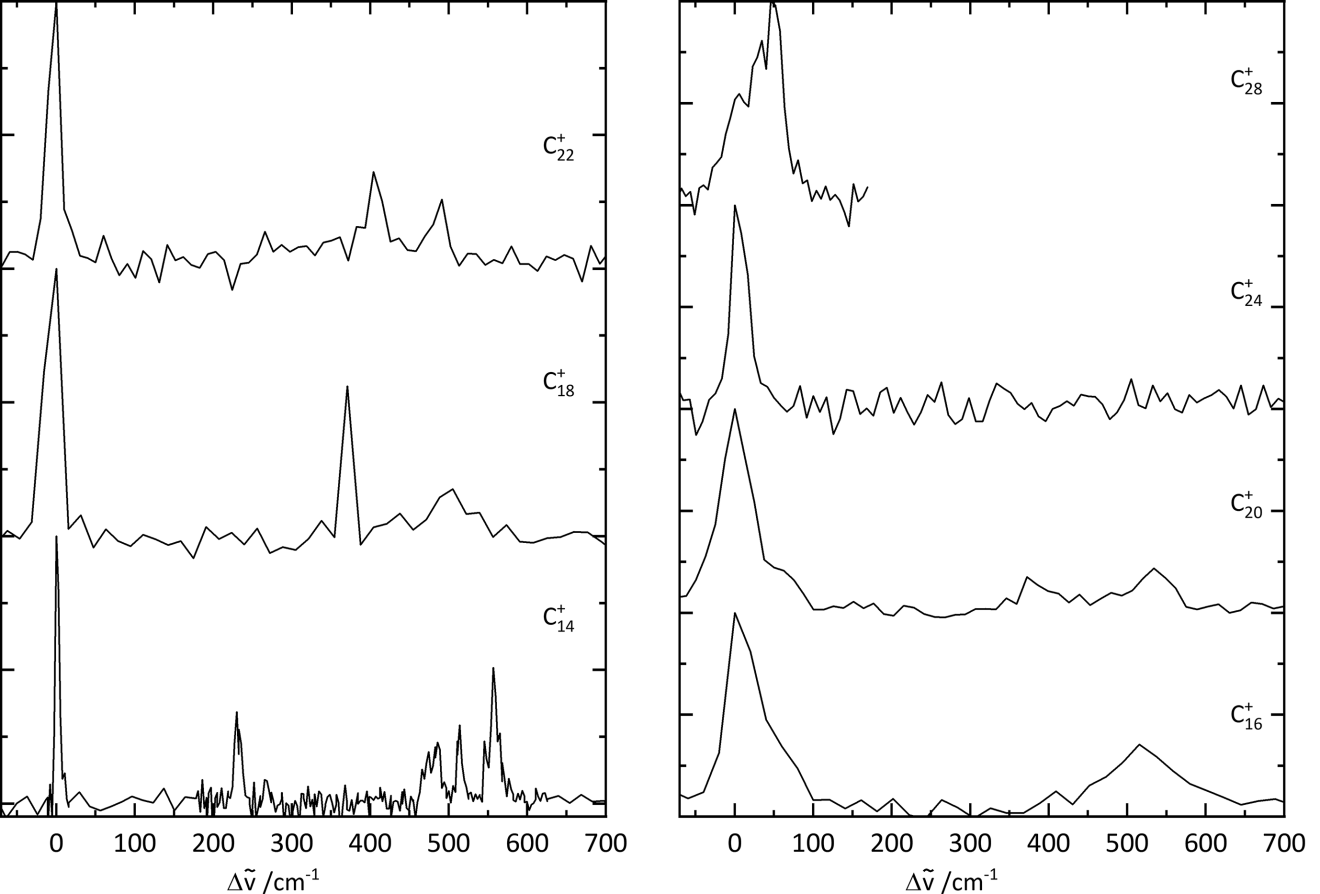
Electronic spectra of monocyclic cations plotted as a function of energy Δν̅ from the origin band. The data are separated into 4*n* + 2 (left) and 4*n* (right) series.

To compare the electronic spectra of the C_2*n*_^+^–He rings, they are plotted in Figure 3. A general trend reported in ref (25) is that the 4*n* + 2 monocyclic cations show narrower (fwhm ≃ 10–20 cm^–1^) absorptions than the 4*n* series (fwhm > 100 cm^–1^). These two groups are expected to differ in terms of their aromatic/antiaromatic nature. Buntine et al. suggest a possible reason for the narrow/broad fwhm variation of the C_4*n*+2_^+^/C_4*n*_^+^ series with reference to the DFT calculations reported by Giuffreda et al.^27^ In particular, it was noted that a quartet excited state lies just above the doublet ground electronic state for the C_4*n*_^+^ ions but higher in energy for the C_4*n*+2_^+^ series. Thus, it was suggested that the electronically excited C_4*n*_^+^ may decay through rapid intersystem crossing due to the high density of quartet vibronic states iso-energetic with the photoexcited doublet, leading to broader widths.^25^

These trends are broadly reflected in the helium tagged C_2*n*_^+^ spectra; however, the distinction between C_4*n*+2_^+^ and C_4*n*_^+^ in terms of width becomes less clear-cut as *n* increases. The fwhm values reported here are determined directly from the measured C_2*n*_^+^ – He data and should be considered as upper limits for the narrower origin bands (e.g., C_14_^+^, C_18_^+^, C_22_^+^) due to the OPO wavelength step size. The width of the origin band of C_14_^+^ of around 6 cm^–1^ would correspond to an excited state lifetime of ∼0.9 ps. The other 4*n* + 2 species observed in the present study, C_18_^+^ and C_22_^+^, have origin bands that are a bit broader and possess similar fwhm’s of around 25 cm^–1^. Interestingly, despite the ability to produce C_26_^+^–He, the monocyclic ring isomer could not be spectroscopically detected, even with the accurate prediction of its origin band wavelength based on Figure 2. This is surprising and based on Table 1, unlikely to be due to a lack of monocyclic cation isomers with *m*/*z* = 312. Rather, it is speculated that changes to the electronic structure and excited state decay mechanisms at this size may be responsible.

On the other hand, the spectra of helium tagged C_4*n*_^+^ rings show some trends. In particular, the fwhm of their origin bands decreases with increasing ring size, with C_12_^+^ being (by far) the broadest. The fwhm’s for C_12_^+^, C_16_^+^, C_20_^+^, C_24_^+^ are 258, 53, 50, 26 cm^–1^, respectively. A decrease in the width of the origin bands with increasing size may be anticipated based on their decreasing rotational constants. However, the fwhm’s obtained in the present study are broader than expected for the extent of the rotational envelope in doublet–doublet transitions of monocyclic rings of this size range (∼2–6 cm^–1^ at 10 K) and are likely to reflect lifetime effects to a greater extent. The shapes of the origin bands in both C_4*n*_^+^ and C_4*n*+2_^+^ spectra are asymmetric and not well reproduced by a single Lorentzian function.

**Figure 4:**
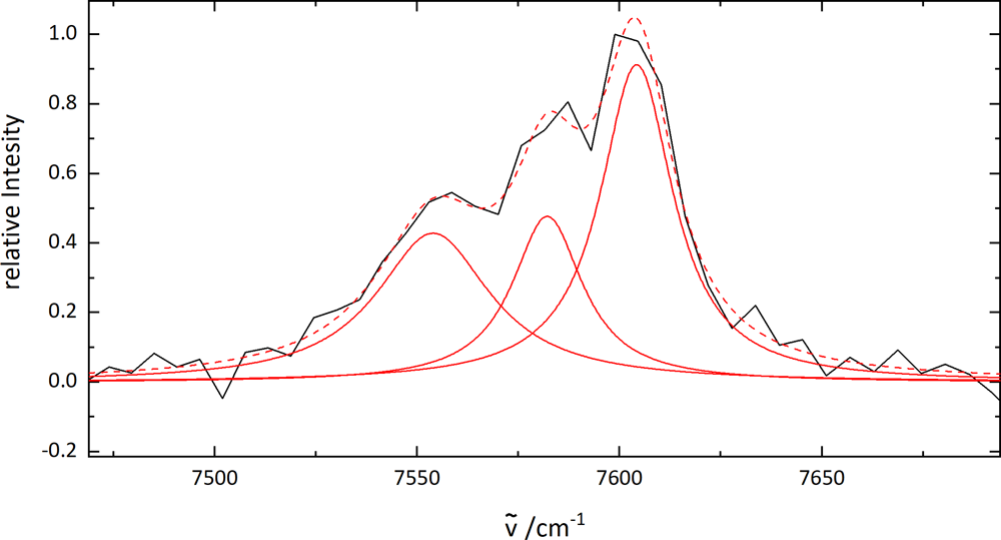
Lowest energy portion of the C_28_^+^–He spectrum. The profile suggests more than one unresolved component and has been fit with three Lorentzian functions (red); the cumulative result is the dotted line.

The lowest energy portion of the of C_28_^+^–He spectrum is shown in Figure 4. The appearance differs somewhat from that of the other 4*n* cations and a fit to the profile suggest the presence of three unresolved features separated by ∼25 cm^–1^. Due to the rather low abundance of monocyclic structures tagged with helium (*m*/*z* = 340), the measured data may show some additional broadening due to saturation. Nevertheless, separation of the envelope into three contributions as shown in Figure 4, suggests a fwhm for the origin of ∼34 cm^–1^ which is more consistent with the values for the other 4*n* species. These absorptions could arise due to excitation of a low-frequency vibrational mode of C_28_^+^ in the excited electronic state or of a mode involving the weakly bound helium atom. It is speculated that the latter explanation, arising from a change in the C_28_^+^···He interaction potential between the ground and excited states may be less likely, as no such patterns are found in the other helium tagged C_2*n*_^+^ spectra. In ref (25) (Supporting Information), harmonic frequencies of C_20_^+^ were reported, including the mode ν_54_ = 21 cm^–1^ so it is conceivable that a low frequency ring vibration in the C_28_^+^ excited state is responsible for the pattern.

### On Comparisons with Astronomical Observations

The electronic spectra of monocyclic rings C_2*n*_^+^ (*n* = 6–14) are appealing in terms of potential astronomical detectability for a couple of reasons. First, these (presumably) lowest energy doublet transitions occur in the spectral region containing the majority of DIBs. Second, these low temperature gas phase measurements show that their electronic oscillator strengths are mostly localized in their origin bands, with the remainder contained within only a few, weaker features. This resembles the situation found for the electronic transitions of C_60_^+^ in the near-infrared and contrasts with that of C_70_^+^. In the case of the C_70_^+^ transition around 7959 Å, the electronic band oscillator strength is distributed over tens of absorptions of similar intensity and thus each band lies below the astronomical detection limits even for column densities of 10^13^ cm^–2^.^14^

The widths of the absorptions are also important to consider. The C_4*n*+2_^+^ species have fwhm of 20 Å or below. The narrowest is for C_14_^+^–He, with a fwhm of less than 3 Å near 6124 Å, which should be observable with current échelle spectrographs. Observation of DIBs broader than 6 Å presents challenges using such instrumentation, as discussed in detail in ref (33), where a number of broad DIBs are also listed. Therefore, dedicated searches based on accurate wavelengths are desirable for the other C_4*n*+2_^+^ and C_4*n*_^+^ ions.

**Figure 5:**
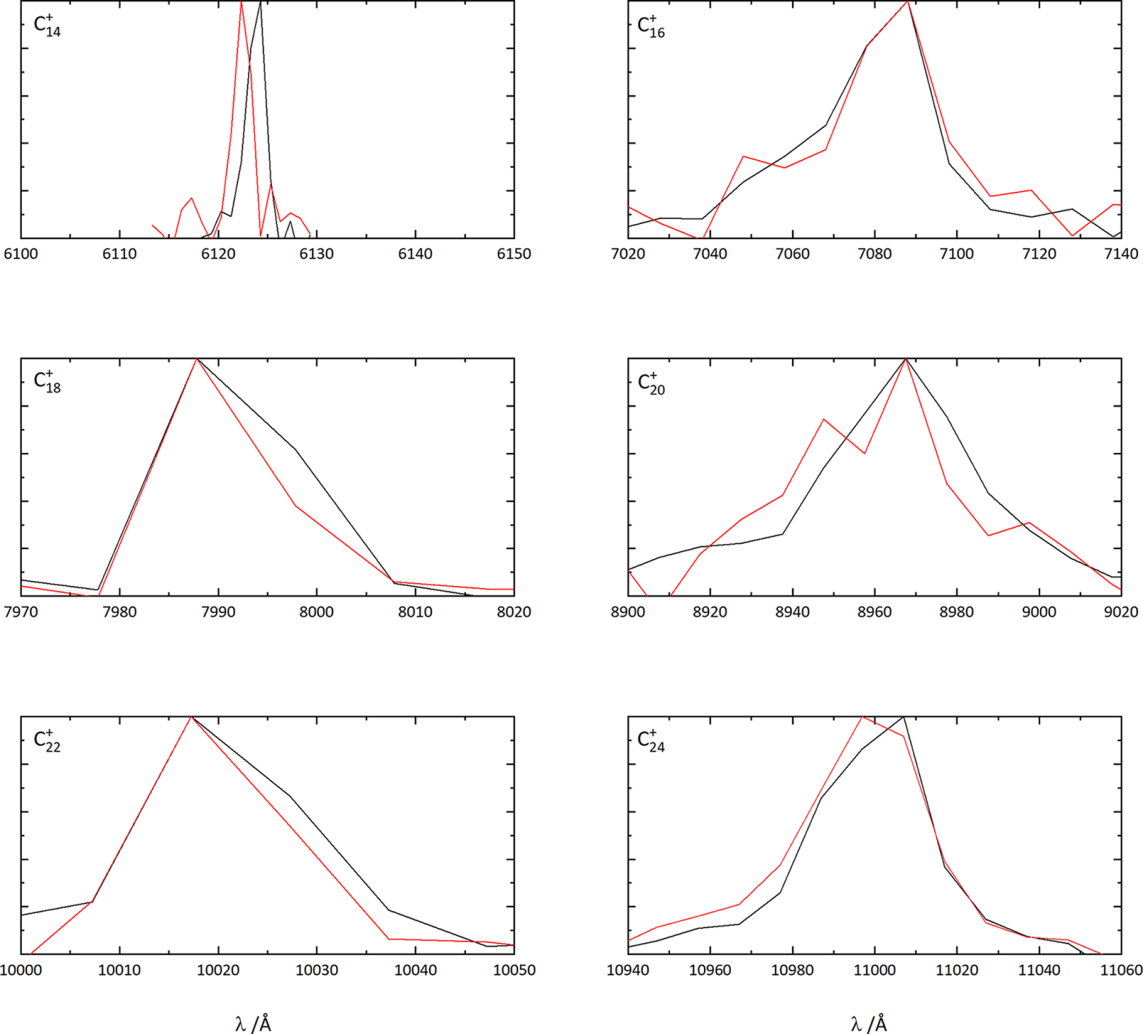
Electronic spectra of monocyclic cations tagged with one (black) and two (red) helium atoms. The data are separated into 4*n* + 2 (left) and 4*n* (right) series.

The absorption wavelengths of the C_2*n*_^+^–He cations presented here will be offset relative to the bare ions due to the presence of the weakly bound atom. For example, in the case of C_60_^+^, this was determined to be less than 1 Å. For smaller carbon cations such as linear C_5_^+^, it was much larger, ∼2.7 Å at 5137 Å corresponding to around 10 cm^–1^. Thus, one may anticipate a shift, depending on the size, of below this value. To gain some insight into this, electronic spectra of C_2*n*_^+^–He_2_ were also acquired and are compared with those of C_2*n*_^+^–He in Figure 5. This indicates a small blue shift of the C_2*n*_^+^–He_2_ absorptions relative to C_2*n*_^+^–He. The wavelengths for C_2*n*_^+^ are thus expected to lie to the red of C_2*n*_^+^–He by less than 6 cm^–1^, and the predicted values for C_2*n*_^+^ are listed in Table 2. The offset applied is determined from the data shown in Figure 5 for C_14_^+^ and C_24_^+^. For the other ions the predicted wavelengths are based on the average (5 cm^–1^) of the He shift for C_14_^+^ (4 cm^–1^) and C_24_^+^ (6 cm^–1^). A possible exception to this trend is C_16_^+^, where a slight red shift may be indicated between C_16_^+^–He and C_16_^+^–He_2_ data in Figure 5. However, it is difficult to make conclusive statements in this case due to a change from the signal to the idler of the used radiation source at 710 nm. The N_2_ tagged spectra reported in Table 1 of ref (25) also indicate a red shift between N_2_ and (N_2_)_2_ using a similar OPO. For more accurate predictions of the narrower bands, higher resolution data are required.

The proximity of the origin band wavelength of C_14_^+^ to DIBs in the visible has been noted in ref (25). The value inferred for the bare ion based on C_14_^+^ – (N_2_)_1,2_ data is 6128 Å, which lies close to the 6126 ± 2 Å predicted here based on helium tagged spectra. The nearby DIB at 6128.20 Å has a fwhm of 2.18 Å,^34^ similar to the width observed for C_14_^+^ – He. With reference to other nonpolar species present in the diffuse ISM (such as C_3_^35^ and H_3_^+^^36^), the monocyclic C_2*n*_^+^ rings may be expected to have rotational temperatures 50–80 K, which is significantly higher than these laboratory measurements. Given the asymmetric profiles of the C_2*n*_^+^–He absorption bands, it would be desirable to obtain more direct measurement of the narrowest C_2*n*_^+^ absorptions through other action spectroscopy methods as recently demonstrated on C_5_^+^,^23^ at elevated temperatures.

## Conclusions

The spectra of C_2*n*_^+^ (*n* = 6–14) are interesting candidates for astrochemical consideration. Their electronic transitions are dominated by strong origin bands and several possess widths below 20 Å in the spectral range of interest for the DIBs. The wavelengths of the C_2*n*_^+^ bands predicted in the present study should guide astronomical searches in the visible/near-infrared. Conclusive assessment of the abundance of these monocyclic ring cations will, however, require a combination of both further laboratory and dedicated observational studies. Laboratory spectra recorded at higher resolution, and exploiting other action spectroscopy methods, are desirable to test the predictions of the C_2*n*_^+^ gas phase wavelengths on the basis of helium extrapolation. Finally, measurement of absorption cross sections or theoretical determination of accurate oscillator strengths, in combination with observational data, is a priority to enable assessment of their abundance in diffuse clouds and thus address the lack of information on the distribution of carbon cation isomers in interstellar environments.
